# Comparison of combined deep and superficial serratus anterior block with thoracic paravertebral block for postoperative pain in patients undergoing video-assisted thoracoscopic surgery

**DOI:** 10.55730/1300-0144.5881

**Published:** 2024-08-04

**Authors:** Gülay ÜLGER, Musa ZENGİN, Onur KÜÇÜK, Ramazan BALDEMİR, Oya KAYBAL, Mehtap TUNÇ, Hilal SAZAK, Ali ALAGÖZ

**Affiliations:** 1 Department of Anesthesiology and Reanimation, Ankara Atatürk Sanatoryum Training and Research Hospital, University of Health Sciences, Ankara, Turkiye; 2 Department of Anesthesiology and Reanimation, Ankara Etlik City Hospital, University of Health Sciences, Ankara, Turkiye

**Keywords:** Postoperative pain, serratus anterior block, thoracic paravertebral block, video-assisted thoracic surgery, visual analogue scale

## Abstract

**Background/aim:**

Thoracic paravertebral block (TPVB) is a well-established procedure for the management of postoperative pain in patients undergoing video-assisted thoracic surgery (VATS). In recent years, there have been studies suggesting that fascial plane blocks may be an alternative to TPVB. The objective of our study was to determine the efficacy of combined deep and superficial serratus anterior block (C-SAPB) as an alternative to TPVB in the management of postoperative analgesia in VATS.

**Materials and methods:**

The patients were divided into two groups: the TPVB group and the C-SAPB group. Both groups were administered the same dose of local anesthetics. Multimodal analgesia was achieved for the groups. The primary outcome measure was visual analog scale (VAS) pain scores recorded within the first 48 h of the postoperative period in each group. The secondary outcomes were analgesic requirement, rescue analgesics, complications rate, and postoperative patient satisfaction.

**Results:**

Thirty patients with C-SAPB and 30 patients with TPVB were analysed. VAS rest and VAS coughing scores were similar between the groups (p > 0.05). Demographic and side effect conditions, total morphine use, additional analgesic needs, vital parameters, block procedure time, and patient satisfaction were also similar between the groups (p > 0.05). Additionally, although block application times were comparable, the time was slightly shorter in C-SAPB.

**Conclusion:**

Similar analgesic efficacy was observed between C-SAPB and TPVB. TPVB maintains its place among the first choices in VATS. The efficacy of C-SAPB is comparable to that of TPVB. While the duration of C-SAPB application is not a significant factor, the brief nature of the procedure and its straightforward administration suggest that it may be an effective method.

## 1. Introduction

Video-assisted thoracic surgery (VATS) is advantageous due to its minimal invasive nature compared with thoracotomy. Postoperative pain is frequent in patients undergoing VATS, although it is less severe than the pain after thoracotomy [1,2]. Postoperative pain may cause increased secretion due to difficulty in coughing. This can lead to complications such as infection, prolonged hospital stays, and long-term pain [3]. Poorly managed acute pain turns into chronic pain and causes patient satisfaction to decrease, and therefore effective control of acute postoperative pain is required [4,5].

The increasing use of ultrasound in various blocks of thoracic surgery has made these applications an effective part of multimodal pain management to relieve acute postoperative pain. The thoracic paravertebral block (TPVB), which is one of these blocks, is performed under ultrasound guidance, is frequently used, and is considered effective [6]. However, experience may be required due to the proximity of the area to be blocked to the pleura and its depth [7,8]. Different studies have shown that TPVB is comparable with epidural analgesia in acute pain management [9]. In VATS, superficial and more easily applied blocks such as erector spinae plane block (ESPB) and serratus anterior plane block (SAPB) have begun to be used increasingly [10]. The purpose of the SAPB application is to block the lateral branches of the intercostal nerves between levels T2 and T9 [11]. SAPB is applied in two ways: under the serratus anterior (deep SAPB) or between the serratus anterior and the latissimus dorsi (superficial SAPB) [12]. However, there are very few studies in the literature in which these two regions are applied in combination for SAPB [13,14].

The combination of analgesic drugs with multimodal techniques has the potential to enhance the efficacy of analgesia in patients. Regional analgesia techniques represent a crucial component of multimodal analgesia techniques employed in VATS. One technique of regional analgesia, TPVB, is recommended and frequently used in VATS. Given the increasing use of fascial plane blocks in recent years, it can be postulated that combined SAPB (C-SAPB) may represent a viable alternative to TPVB. Accordingly, our study was designed with the hypothesis that combining the mechanisms of action of deep and superficial SAPB by considering the multimodal analgesia method may be an alternative to TPVB in postoperative analgesia management. The objective of the present study was to compare the acute postoperative analgesic effect of TPVB application with that of C-SAPB in VATS.

## 2. Materials and methods

### 2.1. Study design and patients

This prospective randomized double-blind equivalence study was performed after approval was obtained from the ethics committee (Date: 09 February 2022, No: E.Kurul-E1-22-2372) and registration at clinicaltrials.gov (Reference number: NCT05255562). Admission of patients to the study started after approval from the ethics committee and clinicaltrials.gov. All patients enrolled in the study gave verbal and written consent. In addition, the patients were given detailed information about VAS and patient-controlled analgesia (PCA) during the preoperative evaluation. The inclusion criteria were as follows: age between 18 and 65 years, American Society of Anesthesiologists (ASA) I–III physical status, body mass index (BMI) 18–30 kg/m^2^, and undergoing elective VATS for lung resections between February 2022 and April 2023.

**Figure 1:**
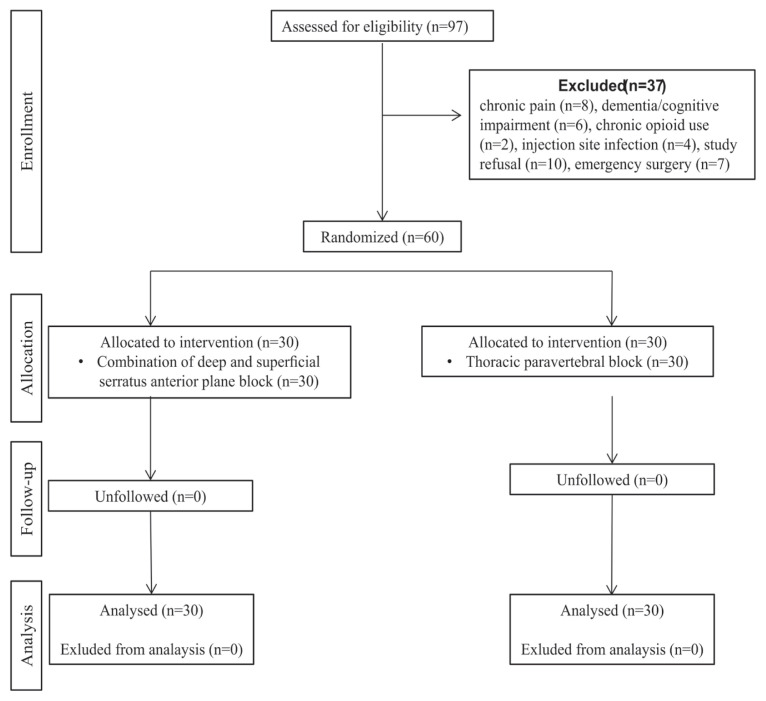
Flow diagram of the participants (Consolidated Standards of Reporting Trials).

Patients were excluded who had undergone emergency operations, received chronic opioid therapy, or had dementia/cognitive impairment, coagulopathy, local infection at the injection site, allergy to the study drugs, or conversion to thoracotomy. The Consolidated Standards of Reporting Trials (CONSORT) checklist was used for the registration and distribution of patients and is shown in Figure 1.

### 2.2. General anaesthesia

All patients received premedication using IV midazolam at a dose of 0.03 mg/kg. After preoxygenation, standard anesthesia induction was performed using fentanyl 1.5 μg/kg, propofol 2 mg/kg, and vecuronium 0.1 mg/kg. A sevoflurane/oxygen/air mixture and 0.01–0.20 μg/kg/min (according to hemodynamic parameters) remifentanil were used for anesthesia maintenance. All patients underwent biportal VATS and a single chest tube was inserted.

### 2.3. Block interventions

Before skin incision, to increase patient comfort and preemptive analgesic effect, block procedures were performed under general anaesthesia. After strict skin antisepsis, a group of experienced anaesthesiologists performed the blocks.

In both groups, a 6–18 MHz transducer (SonoHealth Guangzhou, SonoHealth Medical Technologies Co. Ltd., China) within a sterile drape was used and a 22-gauge and 80-mm nerve block needle (Pajunk, SonoPlexSTIM, Germany) was inserted.

#### 2.3.1. C-SAPB group

The linear transducer was placed on the fifth rib in the mid-axillary region. After the muscle structures were visualized, the needle was advanced up to the fifth rib, under the serratus anterior, using the in-plane technique. Sodium chloride solution was injected to confirm the insertion site and after confirmation bupivacaine 0.25% 15 mL was injected into this area.

After that, the needle was withdrawn around 10 mm and advanced between the serratus anterior and latissimus dorsi. After confirming the location accuracy in this area, bupivacaine 0.25% 15 mL was injected.

#### 2.3.2. TPVB group

The linear transducer was placed at the level of the fifth thoracic vertebrae spinous process and the transverse process was visualized. After identifying the muscle structures, paravertebral space, and pleura, the needle was advanced using the in-plane technique, and bupivacaine 0.25% 30 mL was injected into the paravertebral space.

### 2.4. Analgesia protocol

Before skin closure, dexketoprofen 50 mg and tramadol 100 mg IV were administered. Standard antinausea/vomiting medication (metoclopramide) was administered to all patients. Intravenous morphine PCA (a 1 mg bolus dose of morphine, with a maximum total dose of 16 mg of morphine in 4 h, and a lockout period of 15 min) was administered for 24 h postoperatively.

The patients were monitored in the postoperative care unit for the first 24 h after the surgery. During this period, in addition to morphine PCA, paracetamol 1 g IV 8 hourly and dexketoprofen 50 mg IV 12 hourly were administered. The pain evaluation was performed by visual analog scale (VAS) score (0 = no pain and 100 mm = unbearable pain). Moreover, 0.5 mg/kg tramadol IV (as a form of 5 mg and its multiples and not to exceed the calculated dose according to the weight) as given as “rescue analgesia” to those with a VAS at rest score of 4 and above.

After 24 h, the patients in the ward were administered paracetamol 500 mg tablets 8 hourly and dexketoprofen 50 mg tablets 12 hourly. Despite this treatment, the standard additional analgesia protocol with IV tramadol was continued for patients with VAS 4 and above.

VAS rest and coughing scores were evaluated (hours 1, 2, 4, 8, 16, 24, and 48 postoperatively). The total amount of tramadol consumed as an additional analgesic was converted to morphine equivalent (tramadol consumption mg × 0.1 = morphine mg) and added to the morphine consumed by the patient via PCA during the follow-up period.

Patient data such as demographics, diagnosis, ASA status, operation type, operation duration, hemodynamic data during postoperative follow-up periods, mean blood pressure (MBP), oxygen saturation (SpO_2_), side effects (hypotension, allergies, headache, dizziness, hypoventilation, sleepiness, nausea/vomiting, and infection), VAS scores, block procedure time, total morphine consumption, and rescue analgesia were recorded. The patient’s satisfaction level data were also recorded using a numerical scale (3 - very good, 2 - good, and 1 - bad).

Blocks were applied to all patients by anesthesiologists who were experienced in ultrasound and routinely performed blocks in the department. Postoperative monitoring of the patients’ pain levels was carried out by a nurse who was blinded to the study groups. The data were evaluated by an anesthesiologist independent of this team.

### 2.5. Outcome

The primary outcome measures were the VAS pain score recorded periodically during postoperative rest and coughing in the C-SAPB and TPVB groups. The secondary outcome measures were the analgesic requirement including morphine consumption administered by PCA after the patient’s request and/or the use of rescue analgesics within the first 48 h and complications developing at the end of 48 h and postoperative patient satisfaction.

### 2.6. Sample size

The sample size for the study was calculated using the software G*Power, version 3.1.9.6. The effect size used in the calculation was derived from the study conducted by Qui et al. [7], in which single-injection SAPB was compared with TPVB. In their study [7], the mean 24 h resting VAS score for TPVB was 19 ± 11 mm. To test our primary outcome measure, the minimum clinically significant change in pain measured by VAS, as recognized in the literature, was 13 mm [15]. Accordingly, a minimum sample size of 24 was calculated for each treatment arm, with a type-1 error level of 0.05 and a working power of 90%, to detect a difference of at least 13 mm between the C-SAPB and TPVB arms. In order to allow for potential protocol deviations, a 20% margin of error was incorporated for each treatment arm, and it was decided that 30 patients would be included in each treatment arm.

### 2.7. Randomization and blinding

The study comprised two groups: a C-SAPB group and a TPVB group. The two groups each contained 30 patients. Prior to surgery, each patient was randomly assigned an identification number, which was used to collect all data. The sealed envelope method was employed to assign patients to the two groups. The blocks were performed by an experienced anesthesiologist who was not involved in the randomization or data collection processes. All data were collected blindly by a physician other than the one who performed the randomization and administered the block. Following the surgical procedure, the patients were provided with comprehensive information regarding the use of the PCA device and were not prevented from administering medication. They were instructed to press a button on their hand in the event of experiencing pain. The confidentiality of patient data was protected under the Declaration of Helsinki.

### 2.8. Statistical analysis

SPSS for Windows version 22.0 (SPSS Inc., Chicago, IL, USA) was employed for data analyses. The Kolmogorov–Smirnov test was used to detect normality of the distribution of continuous variables. The homogeneity of variances was evaluated using the Levene test. For normal distributions of continuous data, mean ± standard deviation (SD) was used, while for skewed distributions, median (Q1: 25th percentile - Q3: 75th percentile) was used. Additionally, categorical data were presented as percentages. Student’s t-test was used to analyze statistical differences in normally distributed variables. On the other hand, the Mann–Whitney U test was used to compare data that did not show normal distribution. Pearson’s chi-squared or Fisher’s exact test was used to compare categorical variables. A p-value <0.05 was considered statistically significant.

Graphical representations were obtained using Jamovi (version 2.3.21.0, Sydney, Australia).

## 3. Results

Thirty-seven of 97 patients were excluded and the remaining 60 were randomized (Figure 1). Thirty patients who underwent C-SAPB and 30 patients who underwent TPVB were analyzed. Age, sex distribution, BMI, ASA status, comorbidities, diagnosis, block procedure time, surgery type, and surgery time according to the groups are shown in Table 1 and these data was comparable between the groups (p > 0.05).

**Figure 2:**
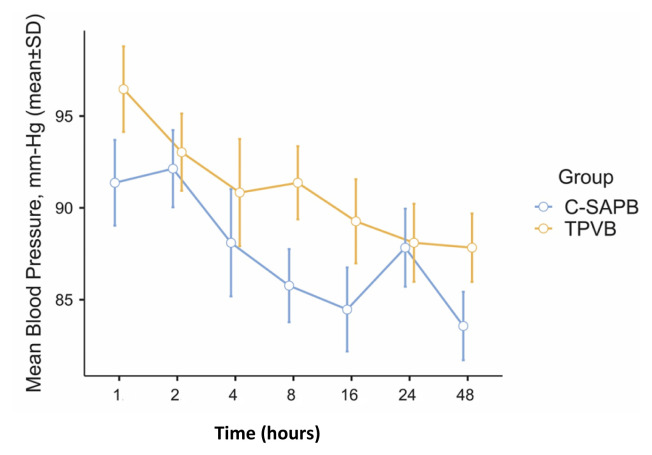
Standard error graph of mean arterial pressure at time points between the groups (C-SAPB: combination of deep and superficial serratus anterior plane block; TPVB: thoracic paravertebral block).

**Figure 3:**
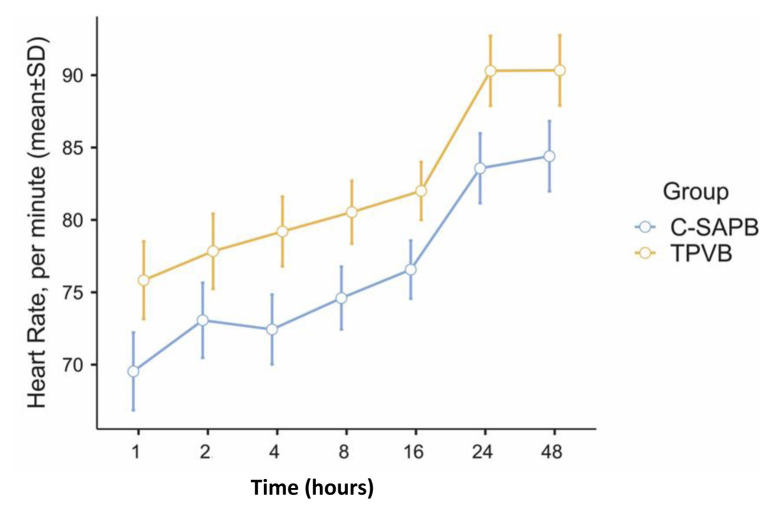
Standard error graph of peak heart rate at time points between the groups (C-SAPB: combination of deep and superficial serratus anterior plane block; TPVB: thoracic paravertebral block).

When the MBP, heart rate, and SpO_2_ values at different periods following surgery were evaluated, these results were also comparable between the groups (p > 0.05, as shown in Figures 2 and 3).

**Figure 4:**
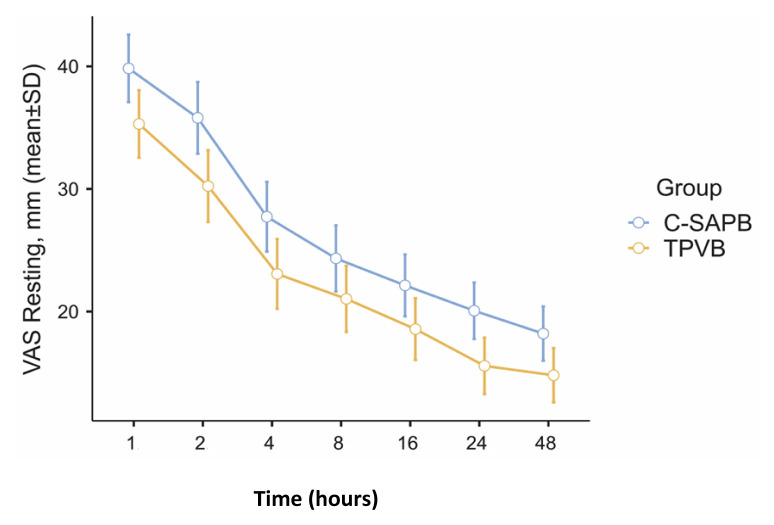
VAS resting error graphs at time points between the groups (C-SAPB: combination of deep and superficial serratus anterior plane block; TPVB: thoracic paravertebral block).

No statistically significant differences were observed when VAS rest scores were examined between the groups (1 h, p = 0.261; 2 h, p = 0.208; 4 h, p = 0.254; 8 h, p = 0.358; 16 h, p = 0.144; 24 h, p = 0.193; 48 h, p = 0.448). Similar comparable results were observed when VAS coughing scores were examined (1 h, p = 0.211; 2 h, p = 0.141; 4 h, p = 0.277; 8 h, p = 0.287; 16 h, p = 0.424; 24 h, p = 0.255; 48 h, p = 0.491) (Table 2). Figures 3 and 4 display the error plots for VAS rest and VAS cough scores between the groups at different time points.

**Figure 5:**
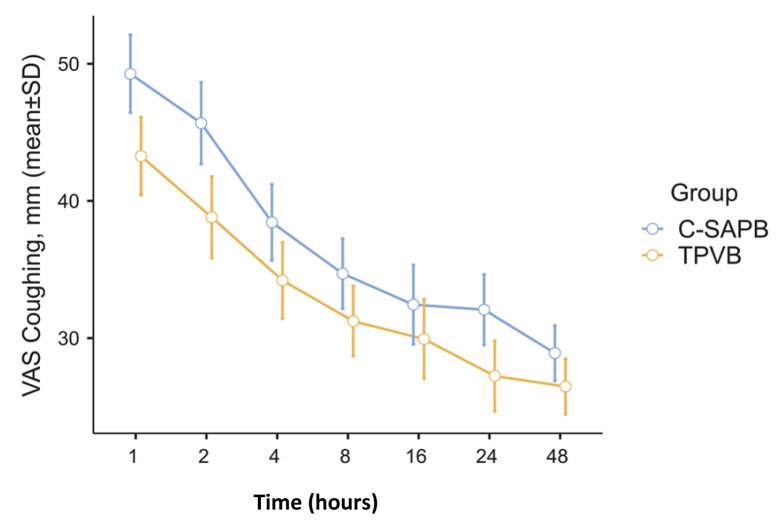
VAS coughing error graphs at time points between the groups (C-SAPB: combination of deep and superficial serratus anterior plane block; TPVB: thoracic paravertebral block).

The amount of morphine requested by PCA after surgery was 21.50 ± 13.02 mg on average in C-SAPB and 22.67 ± 14.09 mg on average in TPVB. No statistically significant difference was observed between the two groups for postoperative morphine demand by PCA (p = 0.740, Table 3). When additional analgesia requests between the groups were examined, 18 (60%) patients in the C-SAPB group and 15 (50%) patients in the TPVB group requested additional analgesia within 48 h of the operation (p = 0.436). The amount of additional analgesia, tramadol demand, between the groups was a median of 32.5 mg (0–90) in the C-SAPB group and a median of 15 mg (0–70) in the TPVB group. Similarly, the amount of additional analgesia consumption between the groups was not statistically significant (p = 0.198). The total morphine consumption within 48 h was 26.85 ± 15.15 mg in the C-SAPB group and 26.16 ± 16.12 mg in the TPVB group. There was no statistically significant difference between the groups in terms of total morphine consumption within 48 h (p = 0.866, Figure 5, Table 3).

Side effects observed at the end of the 48 h postoperatively and patient satisfaction are shown in Table 3. Nausea/vomiting was observed in 5 patients in the C-SAPB group, while it was observed in 3 patients in the TPVB group. Additionally, dizziness was observed in 2 patients in the TPVB group (p = 0.476). Finally, patient satisfaction was similar between the groups after 24 h of follow-up (p = 0.371).

## 4. Discussion

In the present study, we compared ultrasound-guided TPVB and C-SAPB block methods, as a part of multimodal pain therapy, in patients who underwent lung resection. The vital parameters, postoperative analgesic effectiveness, morphine consumption, patients’ side effects, and satisfaction results were similar. Additionally, although block application time was comparable, it was slightly shorter in C-SAPB.

Due to the widespread use of minimally invasive procedures in thoracic surgery in recent years, studies have reported promising results such as a decrease in postoperative pulmonary complications and shorter hospital stays [16]. VATS, compared to thoracotomy, reduces surgical stress and postoperative pain because of its less invasive nature. However, VATS can still cause severe early and long-term pain [17,18]. Postoperative pain management for VATS enables the reduction of postoperative complications and is therefore as important as pain management after open surgery [18]. However, determining effective analgesia management is still an important research topic. Poorly managed analgesia may undesirably affect patients’ quality of life after surgery [18]. For this purpose, the Procedure Specific Postoperative Pain Management (PROSPECT) guideline for pain management after VATS has been published [19].

PROSPECT is a guideline for clinicians to provide supporting information, based on the literature, for postoperative pain management. Among the regional analgesia recommendations for pain management in patients undergoing VATS in the PROSPECT guideline, the first one suggested is TPVB or ESPB [19]. These methods can be applied via a single injection or preferably via a continuous infusion of local anesthetic (LA) through a catheter [19]. In the proposal, SAPB is presented as the second option [19]. However, there is currently no clear suggestion regarding whether to use deep or superficial blocking for SAPB. In the present study, we applied ultrasound-guided TPVB and C-SAPB, as part of multimodal analgesia, for postoperative analgesia in patients who underwent lung resection with VATS. We chose combined superficial and deep application for the SAPB procedure. We aimed to benefit from the advantages of multisite injection with the combined method.

Although TPVB, which has been extensively used in thoracic surgery over the past 3 decades, remains in use in VATS, superficial thoracic fascial plane blocks have become popular due to their ease of application and limited complication rates. However, novel studies are still needed on this subject, and their superiority over each other is still a topic of debate [20].

After thoracic paravertebral injection, LA may remain localized at the injected level but spread superiorly and inferiorly to adjacent levels, laterally into the intercostal space, medially into the epidural space, or a combination of these. It can affect somatic and sympathetic nerves on the same side, including the posterior primary ramus, in several adjacent thoracic dermatomes [21,22]. Considering all these sites of action, it may not be surprising that TPVB provides comprehensive postoperative analgesia. Studies indicate that an analgesic effect similar to that of thoracic epidural analgesia can be achieved with TPVB [23–25]. Although it is performed under ultrasound guidance, complications such as pleural puncture, vascular injury, and even progression to total spinal block may develop in TPVB applications [26]. Additionally, its proximity to vascular neural structures may require more experienced practitioners [26].

Fascial thoracic body blocks are easy to apply because they are far from vascular and neural structures and are more superficial. SAPB, one of these blocks, can be applied superficially or deeply. In deep SAPB application, LA provides analgesia by blocking the anterior and lateral cutaneous branches of the thoracic intercostal nerves [27,28]. The superficial application of SAPB is known to block the anterior and lateral cutaneous branches of the thoracic intercostal nerves and the thoracic longus nerve [29]. A study stated that, in patients who underwent VATS, VAS scores were significantly lower in the SAPB and TPVB groups in the early acute postoperative period compared to the control group [30]. Additionally, there was no significant difference in VAS scores between the SAPB and TPVB groups. In a prospective study comparing TPVB and deep SAPB, similar results were obtained between the two groups in the first 24 h [8]. In the present study, we observed similar analgesic results in the two groups after VATS. Considering the side effects, we can conclude that TPVB and C-SAPB may be applied safely.

The advantages of fascial plane blocks include the ability to easily visualize the application area under ultrasound guidance and faster block application. Baytar et al. [8] observed similar analgesic effects and side effects in a prospective study in which they performed TPVB and deep SAPB in VATS cases. They also found that the procedure time was significantly shorter in patients who underwent deep SAPB [8]. Although similar results were obtained for application times, this period was shorter in C-SABP. This can be explained by the fact that in combined block application, even though there is a single injection, blocks are applied in two different areas.

Many factors can affect LA spread in plane blocks. These factors include volume, applied level, preferred block, and individual anatomical variability [31]. While there are studies comparing different superficial blocks, the use of combined blocks is limited. It has been shown that multisite injection and the techniques involving LA injections into different areas from a single injection site are becoming increasingly recommended. For this purpose, there are combined TPVB–ESPB [32,33] and, albeit limited, C-SAPB [34] applications in the literature. In multisite applications, the aim may be to limit the factors affecting the LA spread mentioned above. Additionally, in case of failure, it may be possible for the other site to act as a backup. Furthermore, the easy applicability of C-SAPB may support VATS as a good option. In our study, in addition to analgesic results like TPVB, the shorter application time, although not significant, suggests that C-SAPB can be an effective method.

Local anesthetic systemic toxicity is one of the most important problems in regional LA applications and it is important to detect findings early and intervene [35]. The limits of LA spread in application areas in plane blocks cannot be clearly explained [36]. Depending on the type of block applied, fascia thickness is an important determinant. While the diffusion of LA is slower in the aponeurotic fascia, diffusion from this area may be faster because the epimysial fascia is thinner [37]. Although there are studies indicating that LA spread over a wider area [20], especially in superficial SAPB, the epimysial nature of the fascia in this area may suggest that systemic spread through thin fascia may be greater. In our study, blocks were performed immediately before skin incision to benefit from the preemptive effect and not to negatively affect patient comfort. Due to this reason, we did not have the opportunity to evaluate the systemic toxicity of LA in patients. However, although not significant, the suppression in both MBP and heart rate in the C-SAPB group may have been due to the LA effect. LA can cause concentration-dependent depression due to the myocardial depression effect [35]. It is also known that this effect suppresses cardiac conduction and contractility in more potent LA agents at significantly lower concentrations than in less effective LA drugs. Although there are limited and not meaningful data, this situation should be taken into consideration, especially in blocks applied to epimysial fascias with potent LA. In this regard, large-series studies measuring LA plasma levels can provide more information.

Another interesting point is that superficial SAPB, particularly applied at high LA volumes, also blocks the thoracic longus nerve and causes a winged scapula [29]. While the thoracodorsal artery can be visualized in ultrasound imaging, blockage of the thoracic longus nerve may not be predictable in patients. This unpleasant clinical situation can be limited by reducing the LA dose and volume in superficial SAPB. In our study, there was no such problem in the postoperative period. Although a total of 30 mL of local anesthetic was used in the combined block, the local anesthetic dose and volume applied to the superficial area was 15 mL. As a result, similar analgesic effects were achieved and the fact that winged scapula side effects were not observed can be considered an advantage.

Our study has some limitations. It was performed in a single center and there was no control group. Secondly, general anesthesia administration prior to the surgical incision to reduce anxiety in patients prevented us from investigating the effectiveness of the block. Moreover, the chronic pain of the patients was not evaluated. Furthermore, the study was completed in a tertiary thoracic surgery hospital, which limits the adaptation of our study to the general population. Finally, the primary aim of our study was to compare the VAS values between the two groups at rest 24 h postoperatively. The 24-h VAS rest values were used to determine the study’s sample size. Since the two blocks applied provide effective and safe postoperative analgesia, the secondary error margin in comparisons between the two groups is high due to the small sample size.

## 5. Conclusion

Although there are ongoing studies on thoracic superficial plane block, there is no optimal application method in this regard, and the volume and dose to be applied remain unclear. In addition, LA injection applications into areas other than multisite or single injection, which have become particularly popular in recent years, also raise questions. The fact that the C-SAPB application is similar to TPVB, which is among the first choices in VATS applications, and has a shorter application time, although not significantly, shows that it will be an effective method. We think that randomized controlled studies are needed on this subject.

## Figures and Tables

**Figure 6:**
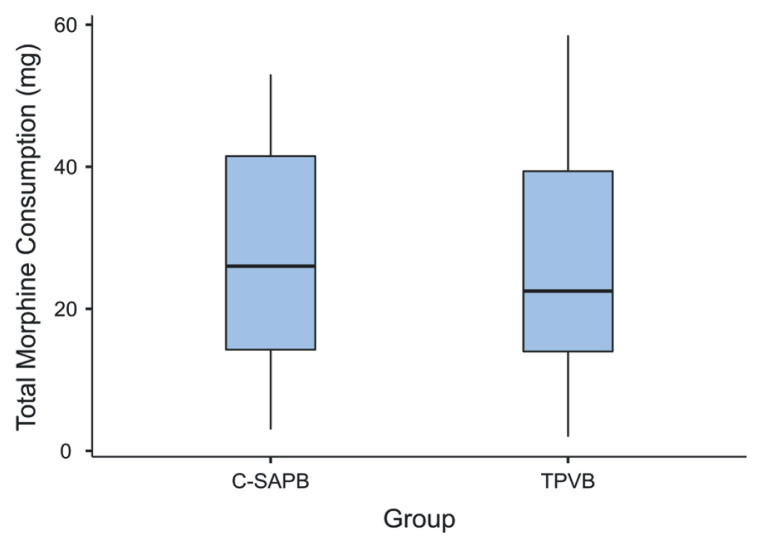
Comparison of 48-h total morphine consumption between the groups (C-SAPB: combination of deep and superficial serratus anterior plane block; TPVB: thoracic paravertebral block).

**Table 1:** Comparison of the demographic data of the groups.

	C-SAPB (n = 30)	TPVB (n = 30)	p-value
**Age, years, median (Q1–Q3)**	56 (40–65)	46 (33–60)	0.152β
**Sex, n (%)**			0.791δ
Female	11 (36.7%)	12 (40.0%)	
Male	19 (63.3%)	18 (60.0%)	
**BMI, kg/m** **^2^** **, median (Q1–Q3)**	26.30 (21.79–29.06)	27.44 (23.53–29.35)	0.399β
ASA, n (%)			0.764δ
ASA I	1 (3.3%)	3 (10.0%)	
ASA II	20 (66.7%)	18 (60.0%)	
ASA III	9 (30.0%)	9 (30.0%)	
**Comorbidities, n (%)**			0.444δ
No	17 (56.7%)	19 (63.3%)	
Hypertension	2 (6.7%)	3 (10.0%)	
Diabetes mellitus	1 (3.3%)	4 (13.3%)	
Coronary artery disease	3 (10.0%)	1 (3.3%)	
COPD-asthma	4 (13.3%)	2 (6.7%)	
Goiter	3 (10.0%)	1 (3.3%)	
**Diagnosis, n (%)**			0.320δ
Mass, malignancy	24 (80.0%)	20 (66.7%)	
Bronchiectasis	-	1 (3.3%)	
Interstitial lung diseases	1 (3.3%)	-	
Bullous lung +pneumothorax	5 (16.7%)	9 (30.0%)	
**Block Duration, sec, median (Q1–Q3)**	187 (180–300)	240 (180–280)	0.542β
**Surgery, n (%)**			1.000δ
Lobectomy	8 (26.7%)	8 (26.7%)	
Wedge, segmentectomy	22 (73.3%)	22 (73.3%)	
**Duration of surgery, min, mean ± SD**	166.73 ± 86.27	145.0 ± 62.64	0.269*

Continuous variables are expressed as either

* the mean ± standard deviation (SD) or

β the median (Q1; 25th percentile – Q3; 75th percentile), and categorical variables are expressed as either

δ frequency (n) or percentage (%).

Continuous variables were compared with Student’s t-test or the Mann–Whitney U test, while categorical variables were compared using Pearson’s chi-square test or Fisher exact test. ASA: American Society of Anesthesiologists; BMI: Body mass index; C-SAPB: The combination of deep and superficial SAPB; COPD: chronic obstructive pulmonary diseases; min: minute; SD: standard deviation; sec: second, TPVB: The Thoracic Paravertebral block.

**Table 2:** Comparison of the groups’ pain scores and VAS scores.

	C-SAPB (n = 30)	TPVB (n = 30)	p-value
	Median (Q1–Q3)	Median (Q1–Q3)	
VAS rest, mm			
1st hour	39 (31–52)	34 (29–45)	0.261β
2nd hour	36 (24–52)	32 (20–42)	0.208β
4th hour	27 (19–35)	24.5 (13–32)	0.254β
8th hour	24 (14–33)	19.5 (14–31)	0.358β
16th hour	22 (14–30)	15.5 (12–25)	0.144β
24th hour	20.5 (13–28)	15.5 (0–24)	0.193β
48th hour	17.5 (10–25)	14 (10–24)	0.448β
VAS coughing, mm			
1st hour	47 (40–61)	45.5 (34–52)	0.211β
2nd hour	41 (33–61)	41 (26–51)	0.141β
4th hour	37.5 (25–45)	33.5 (25–42)	0.277β
8th hour	32.5 (24–47)	30.5 (21–40)	0.287β
16th hour	33 (21–40)	25.5 (23–36)	0.424β
24th hour	32.5 (22–39)	27 (16–37)	0.255β
48th hour	27 (21–36)	25 (21–31)	0.491β

Continuous variables are expressed as the

β median (Q1; 25th percentile - Q3; 75th percentile).

Continuous variables were compared with the Mann-Whitney U test. C-SAPB: the combination of deep and superficial serratus anterior plane block; mm: millimeters; TPVB: Thoracic Paravertebral block; VAS coughing: Visual analog scale cough; VAS rest: Visual analog scale score at rest.

**Table 3:** Comparison of patient satisfaction and additional analgesic need between the groups.

	C-SAPB (n = 30)	TPVB (n = 30)	p-value
Morphine consumption, mg, mean ± SD	21.50 ± 13.02	22.67 ± 14.09	0.740*
Additional analgesia request, n (%)			0.436δ
None	12 (40%)	15 (50%)	
Yes	18 (60%)	15 (50%)	
Tramadol, mg, Median (Q1–Q3)	32.5 (0–90)	15 (0–70)	0.198β
Total morphine consumption, mg, mean ± SD	26.85 ± 15.15	26.16 ± 16.12	0.866*
Side effects, n (%)			0.476δ
None	25 (83.3%)	25 (83.3%)	
Nausea–vomiting	5 (16.7%)	3 (10.0%)	
Other (dizziness)	–	2 (6.7%)	
Patient satisfaction 24^th^ Hour, n (%)			0.371δ
Not satisfied	-	-	
Medium	9 (30.0%)	6 (20.0%)	
Satisfied	21 (70.0%)	24 (80.0%)	

Continuous variables are expressed as either

* the mean ± standard deviation (SD) or

β the median (Q1; 25th percentile - Q3; 75th percentile), and categorical variables are expressed as either

δ frequency (n) or percentage (%).

Continuous variables were compared with Student’s t-test or the Mann–Whitney U test, while categorical variables were compared using Pearson’s chi-square test or Fisher exact test. C-SAPB: combination of deep and superficial serratus anterior plane block; SD: standard deviation; TPVB: thoracic paravertebral block.
